# Genetic evidence for differential functions of *figla* and *nobox* in zebrafish ovarian differentiation and folliculogenesis

**DOI:** 10.1038/s42003-023-05551-1

**Published:** 2023-11-21

**Authors:** Kun Wu, Yue Zhai, Mingming Qin, Cheng Zhao, Nana Ai, Jianguo He, Wei Ge

**Affiliations:** 1 Department of Biomedical Sciences and Centre of Reproduction, Development and Aging (CRDA), Faculty of Health Sciences, University of Macau, 999078 Taipa, Macau China; 2 School of Marine Sciences, Sun Yat-sen University, 519082 Zhuhai, China; 3 Southern Marine Sciences and Engineering Guangdong Laboratory (Zhuhai), 519082 Zhuhai, China

**Keywords:** Oogenesis, Development

## Abstract

FIGLA and NOBOX are important oocyte-specific transcription factors. Both *figla-/-* and *nobox-/-* mutants showed all-male phenotype in zebrafish due to increased dominance of the male-promoting pathway. The early diversion towards males in these mutants has precluded analysis of their roles in folliculogenesis. In this study, we attenuated the male-promoting pathway by deleting *dmrt1*, a key male-promoting gene, in *figla-/-* and *nobox-/-* fish, which allows a sufficient display of defects in folliculogenesis. Germ cells in *figla-/-;dmrt1*-/- double mutant remained in cysts without forming follicles. In contrast, follicles could form well but exhibited deficient growth in *nobox*-/-*;dmrt1*-/- double mutants. Follicles in *nobox*-/-*;dmrt1*-/- ovary could progress to previtellogenic (PV) stage but failed to enter vitellogenic growth. Such arrest at PV stage suggested a possible deficiency in estrogen signaling. This was supported by lines of evidence in *nobox*-/-*;dmrt1*-/-, including reduced expression of ovarian aromatase (*cyp19a1a*) and level of serum estradiol (E2), regressed genital papilla (female secondary sex characteristics), and more importantly the resumption of vitellogenic growth by E2 treatment. Expression analysis suggested Nobox might regulate *cyp19a1a* by controlling Gdf9 and/or Bmp15. Our discoveries indicate that Figla is essential for ovarian differentiation and follicle formation whereas Nobox is important for driving subsequent follicle development.

## Abstract (web summary)

Figla and Nobox are both important for folliculogenesis in zebrafish. Figla plays an essential role in follicle formation, while Nobox is important for promoting subsequent follicle development.

## Introduction

Sex differentiation is the process of gonadal development from bipotential gonads to testis and ovary, which is often initiated by upstream sex-determining factors^1–4^. In the past two decades, evidence has accumulated that both somatic and germ cells play critical roles in sex differentiation and gametogenesis in the differentiated ovary and testis; however, the exact mechanisms and factors involved remain to be elucidated^1,5,6^. Studies in mammals have led to the view that the somatic cells differentiate first in response to sex-determining signals, which in turn program the differentiation of the germ cells into male or female gametes to form testis and ovary, respectively^7–10^. However, this view has been challenged by some studies in fish models. Evidence from both the zebrafish and medaka models suggests important feminizing roles for the germ cells in gonadal differentiation. In zebrafish, germ line deficient fish generated by either mutation of genes specifically expressed in the germ cells such as the *dead end* gene (*dnd*)^11,12^ or expression of a cellular toxin in germ cells^12^ all developed into males with testis structure, suggesting important roles for germ cells in ovarian differentiation. Similarly, germ cell deficiency induced by morpholino-mediated knockdown or mutation of germ cell-specific genes in medaka fish resulted in sex reversal of genetic females (XX) to males^13,14^. A recent study showed that spermatogenesis could take place in the female gonadal environment (ovary) in the mutant of germ cell-specific *foxl3*, indicating again the importance of germ cell-intrinsic cues for sperm-egg fate decision^15^.

Zebrafish is a well-known model organism in biological, biomedical, and environmental research^6,16–18^. Interestingly, unlike mammals and some other fish species, the domesticated zebrafish strains used in research do not seem to have any master sex-determining genes, such as *Sry* in mammals^19,20^, *dmy/dmrt1Y* in medaka^21,22^ and *amhy* in tilapia^23^. The sex of zebrafish is therefore determined by a polygenic mechanism, involving multiple genes^24,25^. The high plasticity of zebrafish gonadal differentiation, which can be influenced by various internal and external factors, makes it an attractive model for investigating the actions and interactions of different factors, including transcription factors such as the factor in the germline alpha (FIGLA) and newborn ovary homeobox gene (NOBOX).

FIGLA and NOBOX are both oocyte-specific transcription factors that play important roles in promoting ovarian development and oogenesis in vertebrates^26–29^. FIGLA was first identified in mice for regulating the expression of zona pellucida genes^29^. Despite being an oocyte-specific factor, the loss of FIGLA in mice did not affect gonadal differentiation at the embryonic stage; however, it caused an arrest of oogenesis at the diplotene stage of meiosis, resulting in failed cyst breakdown and formation of primordial follicles^30^. NOBOX was also discovered in mice, and it played an important role in the formation of primary follicles^26,31^. Similar to FIGLA, the NOBOX null mice also exhibited female infertility with early loss of primary follicles and defective folliculogenesis, exhibiting signs of premature ovarian failure (POF) or insufficiency (POI)^26,32^. Although the expression of NOBOX can be detected in primordial and growing follicles^31,33^, the early defects in folliculogenesis have limited studies on its roles in late stages.

Both FIGLA (Figla/*figla*) and NOBOX (Nobox/*nobox*) have also been studied in teleosts. In medaka, mutation of *figla* caused defects in germ cell cyst breakdown and therefore follicle formation, similar to that of Figla knockout (KO) mice^14,27,30^. As for Nobox, the follicles in medaka mutant (*nobox*−/−) were arrested at the stage of 300 μm-diameter oocytes in the ovary, indicating an essential role for *nobox* in follicle growth^27^. In zebrafish, the loss of *figla* or *nobox* both resulted in all-male offspring^34,35^. In *figla* mutant (*figla*−/−), the oogenesis was blocked early at the stage of follicle assembly or the transition from cystic prefollicular oocytes at chromatin nucleolar stage (CN, stage IA) to follicular perinucleolar oocytes (PN, stage IB), which was followed by sex reversal to males with normal spermatogenesis, resulting in all-male phenotype^34^. As for Nobox, the ovary was extremely underdeveloped in the mutant (*nobox*−/−). In contrast to the *figla* mutant, early PN follicles at the primary growth (PG) stage could occasionally be observed in the *nobox* mutant; however, these follicles failed to develop further to form functional ovaries and all individuals also underwent sex reversal to become males with normal spermatogenesis^35^. Since both *figla* and *nobox* are members of the female-promoting pathway, their loss would disrupt the equilibrium between the male and female-promoting pathways, leading to testis development. As the sex change occurred quickly in female mutants of *figla* and *nobox* before or shortly after ovarian differentiation due to the high plasticity of gonadal differentiation and relatively dominant male-promoting pathway, it is difficult or impossible to characterize their exact functions in controlling folliculogenesis. This has led us to hypothesize that the female-promoting factors such as *figla* and *nobox* could be better studied for their roles in folliculogenesis if the dominance of the male-promoting pathway is alleviated. We have recently tested this idea with aromatase mutant (*cyp19a1a*−/−) by disrupting the male-promoting gene *dmrt1*^36^.

Aromatase (*cyp19a1a*) and doublesex and mab-3-related transcription factor 1 (*dmrt1*) are important for female and male differentiation, respectively. As in other vertebrates, *cyp19a1a* is essential for the production of estrogens in fish^37^, which are important for female gonadal differentiation^38^. On the other hand, *dmrt1* is a critical male-promoting transcription factor essential for testis development and spermatogenesis^39^. As expected, deletion of the *cyp19a1a* gene in the zebrafish resulted in an all-male phenotype whereas the loss of *dmrt1* gene led to a female-biased sex ratio and underdeveloped testis in males^36,40–43^. Interestingly, our recent study showed that simultaneous disruption of *dmrt1* and *cyp19a1a* rescued the all-male phenotype of the *cyp19a1a* mutant in zebrafish and the follicles in the double mutant (*cyp19a1a−/−;dmrt1−/−*) could develop well up to the previtellogenic (PV) stage (stage II) with normal formation of cortical alveoli but not yolk granules. This observation suggests that the absence of ovaries in the *cyp19a1a−/−* mutant is likely due to the early and quick diversion of females to males via sex reversal, making it difficult to evaluate the roles of estrogens in follicle development. This discovery reveals that estrogens are not essential for early follicle development including PG-PV transition and subsequent growth of PV follicles, but they are important for late stages of vitellogenic growth^36^. This observation supports our view that the female-promoting factors can be better characterized for their roles in controlling folliculogenesis when the male-promoting pathway is attenuated or blocked to delay or prevent the female-to-male sex reversal.

In this study, we attenuated the male-promoting pathway by disrupting *dmrt1* to study the differential roles of Figla and Nobox in controlling zebrafish folliculogenesis. We created two double mutants of *figla* and *nobox* with *dmrt1* (*figla−/−;dmrt1−/−* and *nobox−/−;dmrt1−/−*) with the aim to prevent early female-to-male sex reversal so as to allow *figla−/−* and *nobox−/−* to fully display their developmental defects in ovarian formation and folliculogenesis. Our data provided strong evidence that both Figla and Nobox play important roles in controlling follicle development; however, they work at different time points of ovarian development and folliculogenesis. Figla is mainly involved in controlling cyst breakdown and follicle formation whereas Nobox controls subsequent follicle development including follicle activation (primary growth-secondary growth transition) and vitellogenic growth. Furthermore, the oocyte specific transcription factor Nobox controls vitellogenic growth by regulating aromatase (*cyp19a1a*) expression in the follicle cells, which might be mediated by oocyte-secreted signaling molecules, e.g., Gdf9 and Bmp15.

## Results

### Roles of Figla in follicle formation

Cyst breakdown or follicle assembly is a critical stage in folliculogenesis^44^. We recently demonstrated in zebrafish that the loss of Figla (*figla−/−*) prevented cyst breakdown as no individual follicles could form in the mutant, suggesting an important role for Figla in controlling the transition from the cystic oocytes at prefollicular chromatin nucleolar (CN) stage (stage IA) to individual follicles with perinucleolar (PN) oocytes (stage IB). The mutant showed an all-male phenotype in the end^34^. To further explore the roles of Figla in follicle development, we created a double mutant of *figla* and *dmrt1* genes (*figla*−/−;*dmrt1*−/−) to prevent early sex reversal.

**Fig. 1:**
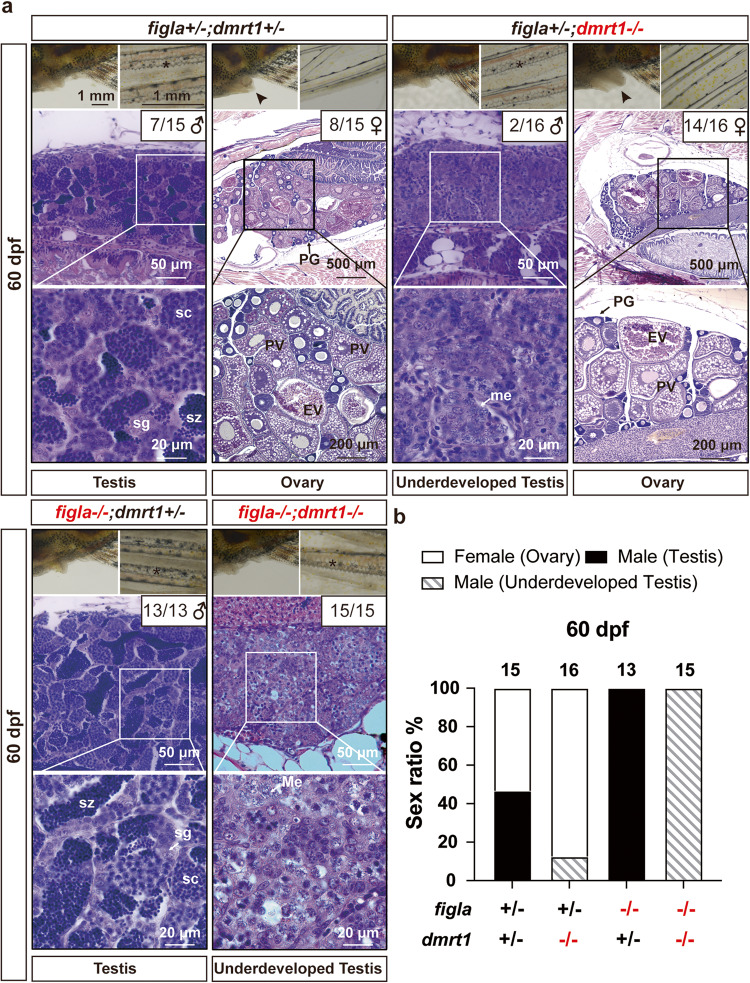
Phenotype analysis of different genotypes of *figla* and *dmrt1* mutations at 60 dpf. **a** Gonads and secondary sex characteristics of four different genotypes: normal ovary and testis in the control (*figla*+/−;*dmrt1*+/−; *n* = 15 independent fish, 7 males, 8 females); normal ovary and underdeveloped testis in *dmrt1* single mutant (*figla*+/−;*dmrt1−/−*; *n* = 16 independent fish, 2 males, 14 females); all-male testis in *figla* single mutant (*figla*−/−;*dmrt*+/−; *n* = 13 independent fish, 13 males) and underdeveloped testis in *figla* and *dmrt1* double mutant (*figla*−/−;*dmrt1*−/−; *n* = 15 independent fish). Asterisk, breeding tubercles on pectoral fins; arrowhead, genital papilla. **b** Sex ratio in four different genotypes at 60 dpf. The sample sizes for independent fish are shown at the top of the columns. PG primary growth, PV previtellogenic, EV early vitellogenic, me meiotic cells, sc spermatocytes, sg spermatogonia, sz spermatozoa.

Histological analysis of gonadal development at 60 dpf showed well-differentiated ovary and testis in the control fish (*figla*+*/−;dmrt1*+*/−*) with normal sex ratio and gametogenesis. In *dmrt1* single mutant (*figla*+*/−;dmrt1−/−*), the sex ratio was biased towards females with normal follicle development; however, the males showed underdeveloped and dysfunctional testis with spermatogenesis blocked at early meiotic stage. As observed previously, all *figla* single mutants (*figla−/−;dmrt1*+*/−*) were males showing normal spermatogenesis. Interestingly, all double mutant fish (*figla−/−*;*dmrt1−/−*) had underdeveloped testis identical with the males of *dmrt1* single mutant. Also, the secondary sex characteristics in *figla* and *dmrt1* double mutants (*figla−/−*;*dmrt1−/−*) were all male-like (with breeding tubercles and without genital papilla) at 60 and 150 dpf (Fig. 1a; Supplementary Fig. 1). Importantly, no follicles could be seen in the gonads of the double mutant at 60 dpf, suggesting failed follicle formation as 60 dpf provided enough time for early follicle formation (Fig. 1). These results suggest that disruption of *dmrt1* could not rescue the female fate of the *figla* mutant as seen with *cyp19a1a* mutant^36^. The gonads could not develop into functional testis or ovary in the absence of *dmrt1* and *figla*, suggesting critical roles for *dmrt1* and *figla* in spermatogenesis and oogenesis, respectively.

### Roles of Nobox in follicle development

Similar to *figla*, knockout of *nobox*, another oocyte-specific transcription factor, also led to an all-male phenotype in zebrafish^35^. However, in contrast to *figla* whose mutation caused complete failure of follicle formation from the cystic oocytes, the follicles could occasionally form and develop into typical PN follicles at early primary growth (PG) stage in *nobox* mutants (*nobox*−/−), more advanced compared to the *figla* mutant (*figla*−/−)^34,35^. However, the mutant ovaries were extremely underdeveloped and the follicles failed to grow further. The mutant female fish soon underwent sex reversal to become males^35^. To prevent gonadal masculinization, we created a double mutant with *dmrt1* mutation to block gonadal masculinization.

**Fig. 2:**
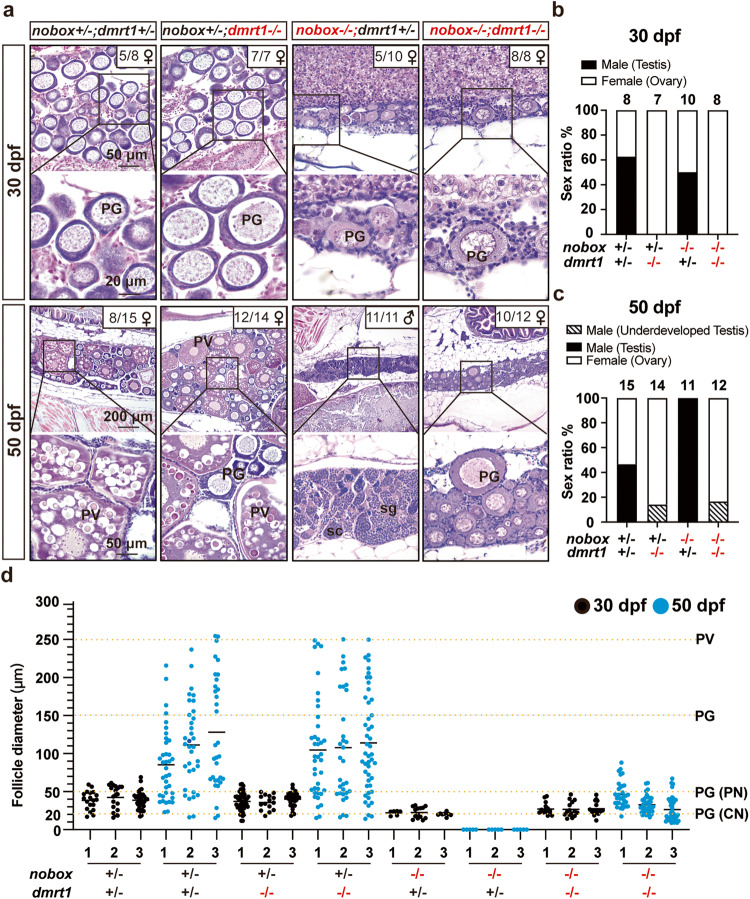
Rescue of the all-male phenotype of *nobox* mutant by simultaneous mutation of *dmrt1*. **a** Gonadal histology of *nobox* mutant and double mutant with *dmrt1* mutation in early gonadal differentiation (30 and 50 dpf). **b** Sex ratio in four different genotypes of *nobox* and *dmrt1* mutations at 30 dpf (*nobox*+*/−*;*dmrt1*+*/−*: *n* = 8 independent fish; *nobox*+*/−*;*dmrt1*−/−: *n* = 7 independent fish; *nobox*−/−;*dmrt1*+*/−*: *n* = 10 independent fish; *nobox*−/−;*dmrt1*−/−: *n* = 8 independent fish). **c** Sex ratio in four different genotypes of *nobox* and *dmrt1* mutations at 50 dpf (*nobox*+*/−*;*dmrt1*+*/−*: *n* = 15 independent fish; *nobox*+*/−*;*dmrt1*−/−: *n* = 14 independent fish; *nobox*−/−;*dmrt1*+*/−*: *n* = 11 independent fish; *nobox*−/−;*dmrt1*−/−: *n* = 12 independent fish). **d** Follicle composition of different genotypes at 30 and 50 dpf (*n* = 3 independent fish). The horizontal black lines represent the mean. PN perinucleolar oocytes, CN chromatin nucleolar oocytes.

At 30 dpf, PN follicles of early PG stage could be observed in the gonads of about 50% *nobox* single mutant fish (*nobox*−/−*;dmrt1*+*/−*; 5 females/10), similar to the female ratio in the control fish (*nobox*+*/−;dmrt1*+*/−*; 5/8, 62.5%); however, compared to the control, the follicles in the *nobox* single mutant were obviously underdeveloped with smaller number and size, suggesting a deficiency in ovarian development (Fig. 2a, b). At 50 dpf, none of the *nobox* single mutant fish contained follicles in the gonads and all fish were males with normal spermatogenesis (11 males/11) as we reported previously^35^, suggesting a quick and early sex reversal from females to males. Interestingly, all double mutant fish (*nobox*−/−*;dmrt1*−/−) had PN follicles at PG stage in their gonads (8/8) at 30 dpf and these follicles seemed better developed than those in the *nobox* single mutant. At 50 dpf, the sex ratio of the double mutant fish remained to be female-biased (10 females/12, 83.3%) with well-formed PG follicles in the ovary, in contrast to the all-male phenotype in *nobox* single mutant (Fig. 2c, d); however, the follicles were all arrested at early PG stage without formation of cortical alveoli, in contrast to those in the control and *dmrt1* single mutant (*nobox*+*/−;dmrt1*−/−) (Fig. 2a, d). These results suggest that the loss of *dmrt1* successfully blocked early sex reversal of *nobox* mutant to males.

**Fig. 3:**
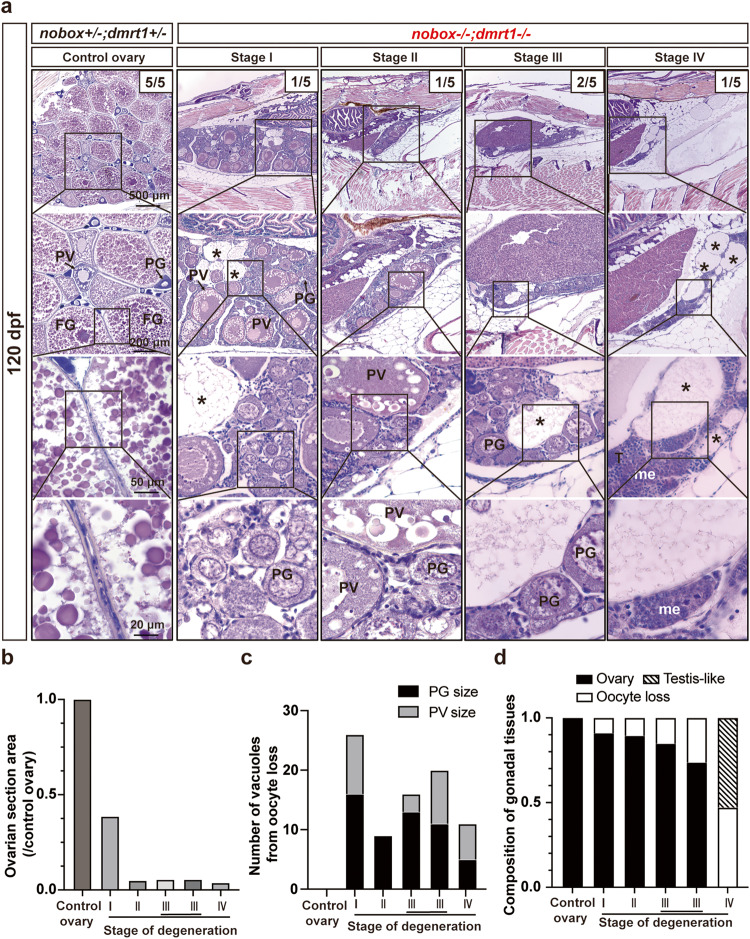
Long-term degeneration of the double mutant ovaries. **a** The ovaries in the double mutant (*nobox*−/−;*dmrt1*−/−) showed different degrees of degeneration at 120 dpf with loss of oocytes and decreased ovarian size (*n* = 5 independent fish). In addition, empty cavities or vacuoles left by degenerated oocytes were often observed (asterisks). The degenerated ovaries were gradually replaced by testicular tissues with meiotic cells (me); however, spermatogenesis could not proceed further due to the lack of *dmrt1*. The ovarian degeneration process was categorized into four stages based on gonadal size and morphological features. In stage I, the ovary was significantly smaller than the control (approximately half the size), containing both PG and PV follicles. Stage II was characterized by a dramatically reduced ovarian size, while still housing PG and PV follicles. In stage III, the ovary contained PG follicles only. In stage IV, the ovary was devoid of all follicles, featuring empty cavities or vacuoles left by degenerated oocytes. Additionally, testicular tissues began to emerge with meiotic germ cells. **b** Ovarian sizes of the control and double mutant ovaries undergoing degeneration. The area size of the largest section of each ovary was measured with ImageJ and the data are expressed as the ratios relative to the control. **c** Number of vacuoles resulting from oocyte loss. The vacuoles were counted on the largest section and classified based on their sizes compared to those of PG and PV follicles. **d** Composition of gonadal tissues in the control and double mutant ovaries. The areas of different gonadal tissues were measured using ImageJ.

To explore the fate of the arrested follicles in the double mutant ovary, we examined the fish at 120 dpf when the control zebrafish were sexually mature with all stages of follicles present in the ovary. Interestingly, we could commonly see follicles that had entered previtellogenic stage (PV, stage II) with abundant cortical alveoli, indicating that the follicles could overcome the arrest and underwent follicle activation (PG-PV transition) in the absence of Nobox (Fig. 3a). This is similar to our observation in the double mutant of *cyp19a1a* and *dmrt1* (*cyp19a1a*−/−*;dmrt1*−/−), but with a significant delay^36^. What should be noted is that while the follicles could develop to the PV stage, the ovaries of the double mutant were smaller than the well-formed ovaries in *nobox*+*/−;dmrt1*+*/−* controls (Fig. 3a, b). The mutant ovaries showed a progressive degeneration with gradual loss of oocytes and a multitude of empty cavities or vacuoles, presumably the remnants of the degenerated or lost oocytes (Fig. 3a, c). The phenotype of oocyte loss was similar to the observation in *Nobox* null mice^26^. The ovarian degeneration was followed by the initiation of sex reversal to males (Fig. 3d). For the convenience of studying and understanding this process, we divided the process of ovarian degeneration in the double mutant into four stages. At Stage I, the ovaries were well formed and structured, containing both PG and PV follicles; however, they were smaller than the control ovaries and contained abundant somatic cells in the interfollicular spaces. Stage II ovaries still contained PG and PV follicles, yet they were markedly smaller in size. At Stage III, the ovaries contained PG follicles only, and at Stage IV, the ovarian tissues had been supplanted by testicular tissues with meiotic cells (Fig. 3).

### Deficiency of estrogen signaling in *nobox* mutant females

The blockade of follicle development at PV stage with normal formation of cortical alveoli but without yolk accumulation in the double mutant oocytes (*nobox*−/−*;dmrt1*−/−) raised an interesting question about the involvement of estrogen signaling as estrogen is essential for vitellogenin production and therefore the transition from PV to early vitellogenic (EV) stage^45,46^. To test this hypothesis, we performed a series of experiments.

**Fig. 4:**
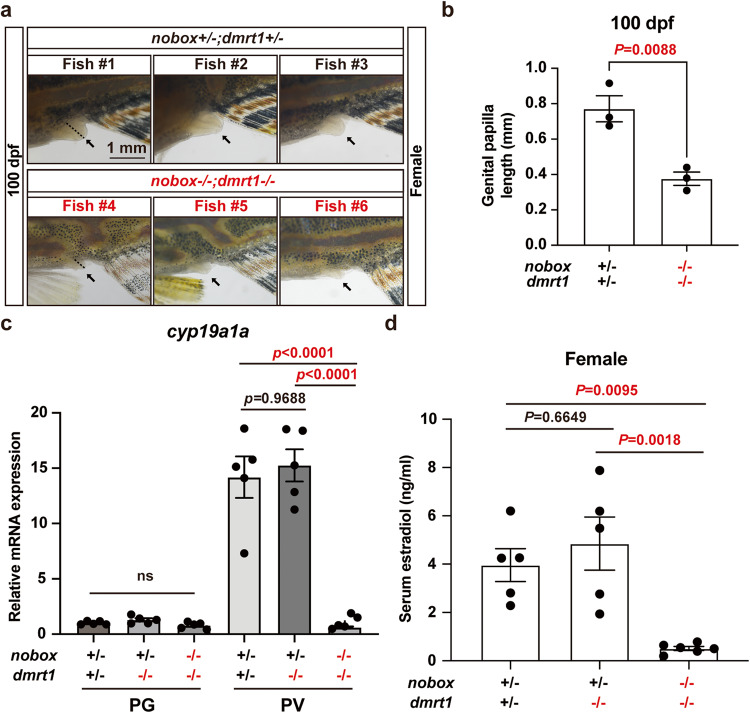
Evidence for estrogen deficiency in double mutant females. **a** Genital papilla in the control (*nobox*+*/−*;*dmrt1*+*/−*; *n* = 3 independent fish) and double mutant (*nobox*−/−;*dmrt1*−/−; *n* = 3 independent fish) females (arrow). The dotted line showed the length of the genital papilla. **b** Length of genital papilla in the controls and double mutant females (*n* = 3 independent fish). Data shown are mean ± SEM (*P* = 0.0088 by unpaired Student’s two-tailed *t* test). **c** Expression of *cyp19a1a* in PG and PV follicles (n = 5 independent samples). Total RNA was extracted from the isolated PG and PV follicles and reverse transcribed into cDNA for real-time PCR analysis. Each data point represents PG or PV follicles isolated and pooled from two fish for each genotype. Data shown are mean ± SEM, *P* values revealed by one-way ANOVA and Tukey’s test. **d** Serum E2 levels in the control (*nobox*+*/−*;*dmrt1*+*/−*; *n* = 5 independent fish), *dmrt1* single mutant (*nobox*+*/−*;*dmrt1*−/−; *n* =5 independent fish) and double mutant (*nobox*−/−; *dmrt1*−/−; *n* = 6 independent fish) females at 100 dpf. Data shown are mean ± SEM (*nobox*+*/−*;*dmrt1*+*/−* v.s *nobox*+*/−*;*dmrt1*−/−: *P* = 0.6649; *nobox*+*/−*;*dmrt1*+*/−* v.s *nobox*−/−;*dmrt1*−/−: *P* = 0.0095; *nobox*+*/−*;*dmrt1*−/− v.s *nobox*−/−;*dmrt1*−/−: *P* = 0.0018; *P* values revealed by one-way ANOVA and Tukey’s test).

**Fig. 5:**
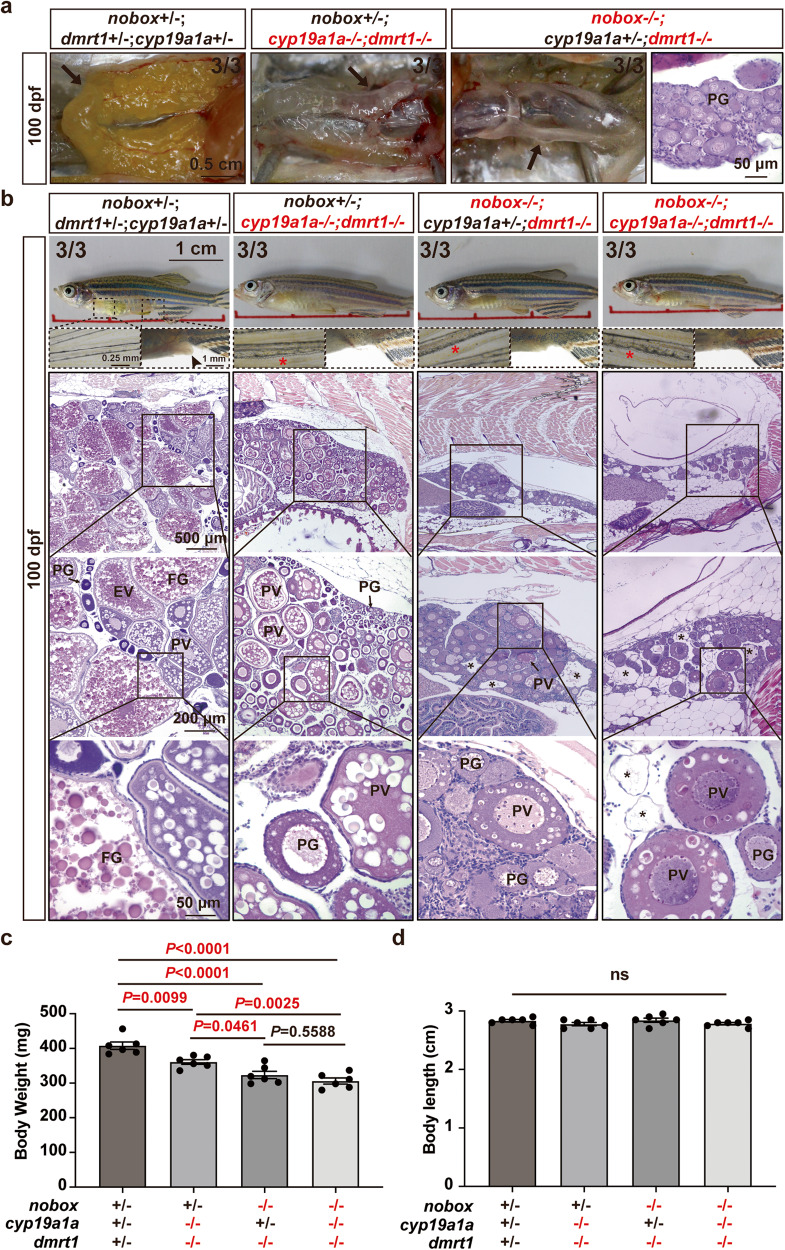
Ovarian growth and follicle development in the triple mutant of *cyp19a1a*, *nobox* and *dmrt1*. **a** Gross morphology of the ovaries (arrow) in females of the control (*nobox*+*/−*;*cyp19a1a*+/−;*dmrt1*+*/−*, *n* = 3 independent fish), *cyp19a1a* and *dmrt1* double mutant (*nobox*+*/−*;*cyp19a1a*−/−;*dmrt1*−/−, *n* = 3 independent fish), and *nobox* and *dmrt1* double mutant (*nobox*−/−;*cyp19a1a*+/−;*dmrt1*−/−, *n* = 3 independent fish). **b** Ovarian histology and secondary sex characteristics in different genotypes: control (*nobox*+*/−*;*cyp19a1a*+/−;*dmrt1*+*/−*, *n* = 3 independent fish), *cyp19a1a* and *dmrt1* double mutant (*nobox*+*/−*;*cyp19a1a*−/−;*dmrt1*−/−, *n* = 3), *nobox* and *dmrt1* double mutant (*nobox*−/−;*cyp19a1a*+/−; *dmrt1*−/−, *n* = 3 independent fish), and *nobox, cyp19a1a* and *dmrt1* triple mutant (*nobox*−/−;*cyp19a1a*−/−; *dmrt1*−/−, *n* = 3 independent fish). Red asterisk, breeding tubercles; arrowhead, genital papilla; black asterisk, vacuoles left by the lost oocytes. **c**, **d** Body weight and body length of the triple mutant of *cyp19a1a*, *nobox* and *dmrt1* (*n* = 6 independent fish). Data shown are mean ± SEM, *P* values revealed by one-way ANOVA and Tukey’s test.

First, we examined the genital papillae at 100 dpf as this female secondary sex characteristic is estrogen-dependent^47,48^. The genital papillae formed well in control females; however, they were significantly regressed in female double mutants (*nobox*−/−*;dmrt1*−/−) with shortened lengths (Fig. 4a, b). Second, we assessed the expression of *cyp19a1a* in the ovary and determined the estradiol (E2) concentrations in the serum of double mutant females. Both the mRNA expression of *cyp19a1a* in PV follicles and serum estradiol level were significantly lower in double mutant females (*nobox*−/−*;dmrt1*−/−) than the control (*nobox*+*/−;dmrt1*+*/−*) and *dmrt1* single mutant (*nobox*+*/−;dmrt1−/−*). There was no significant difference in *cyp19a1a* expression at PG stage, probably because its expression level was very low or barely detectable at this stage^49^ (Fig. 4c, d). These lines of evidence indicate that estrogen synthesis was significantly reduced in the double mutant (*nobox−/−;dmrt1−/−*). Third, we created a triple mutant of *nobox*, *cyp19a1a* and *dmrt1* genes (*nobox−/−;cyp19a1a−/−;dmrt1−/−*). Compared with *cyp19a1a*−/−*;dmrt1*−/−, the ovaries from the double and triple mutants containing *nobox*−/− (*nobox*−/−*;dmrt1*−/− and *nobox*−/−*;cyp19a1a*−/−*;dmrt1*−/−) were generally smaller (Fig. 5a, b), which was also reflected in body weight (Fig. 5c). Histological analysis showed that the triple mutant phenocopied the double mutant (*nobox*−/−*;dmrt1*−/−) at 100 dpf with no additional impact and the follicles could enter PV stage but not vitellogenic stage in both mutants. Although the triple mutant phenocopied the double mutant of *cyp19a1a* and *dmrt1* (*cyp19a1a*−/−*;dmrt1*−/−) in terms of follicle activation characterized with the formation of cortical alveoli, its ovary was underdeveloped with fewer follicles (Fig. 5b). The mutations of these genes did not seem to have any pleiotropic effects as no significant difference was observed in body length (Fig. 5d).

**Fig. 6:**
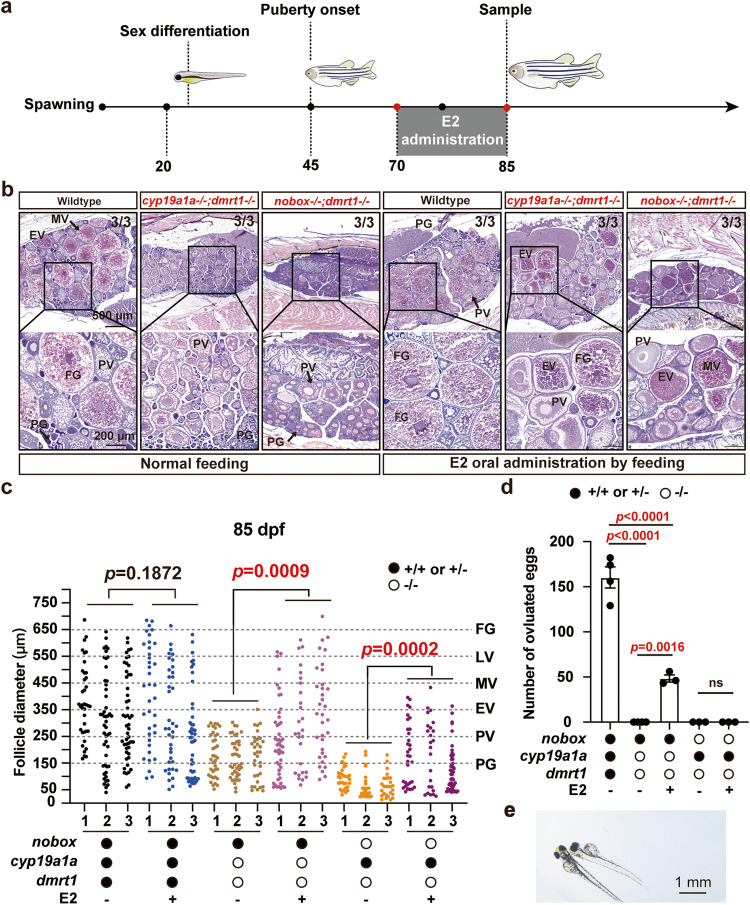
Rescue of vitellogenic growth in the double mutants (*nobox*−/−*;dmrt1*−/− and *cyp19a1a*−/−;*dmrt1*−/−) by E2 treatment. **a** Schematic illustration of E2 treatment. **b** Ovarian histology of different genotypes in the control group with normal feeding and the E2 treatment group fed with an E2-containing diet (*n* = 3 independent fish). Vitellogenic growth characterized with yolk granule accumulation resumed in both double mutants (*nobox*−/−*;dmrt1*−/− and *cyp19a1a*−/−*;dmrt1*−/−), which were blocked at the PV stage with the formation of cortical alveoli but not yolk granules. **c** Follicle composition of different genotypes in different treatment groups (*n* = 3 independent fish). The data points shown are diameters of individual follicles and the statistical significance of the means was demonstrated by unpaired Student’s two-tailed *t* test. **d** Fecundity of different genotypes and treatments. Each data point represents the number of eggs spawned by each female mated with one wild-type male (Control group: *n* = 4 independent experiments; Other groups: n = 3 independent experiments). The sexes of examined females were further confirmed by histology after mating. Data shown are mean ± SEM, *P* values revealed by one-way ANOVA and Tukey’s test. **e** The offspring from E2-treated females of *cyp19a1a*−/−;*dmrt1*−/− double mutant.

Finally, to provide direct and conclusive evidence for the involvement of estrogen signaling in the follicle blockade at PV-EV transition in the double mutant (*nobox*−/−*;dmrt1*−/−), we performed an experiment to treat both double mutants *nobox*−/−*;dmrt1*−/− and *cyp19a1a*−/−*;dmrt1*−/− with E2, the major endocrine hormone that promotes vitellogenesis^50^. To avoid the toxic effects of estrogens on follicle development at high dosage^51,52^, we adopted the treatment scheme optimized in our recent study^52^. The fish were treated with E2 by oral administration of E2-containing diet (2 μg/g) for 15 days from 70 to 85 dpf (Fig. 6a). Histological analysis showed that E2 treatment could rescue the PV-EV blockade with yolk accumulation resumed in both double mutants (Fig. 6b). The follicles in the double mutant *cyp19a1a*−/−*;dmrt1*−/− could develop to the full size of FG stage, similar to the follicles in the control fish. In contrast, although vitellogenic growth also resumed in the double mutant *nobox*−/−*;dmrt1*−/−, the follicles could only grow to the range of mid-vitellogenic (MV) stage (Fig. 6c). In agreement with the histological observations, the E2-treated *cyp19a1a*−/−*;dmrt1*−/− females could spawn to produce fertilizable eggs (Fig. 6d) and live offspring (Fig. 6e); however, the E2-treated *nobox*−/−*;dmrt1*−/− females were infertile (Fig. 6d).

### Potential roles of Gdf9 and Bmp15 in mediating Nobox actions

**Fig. 7:**
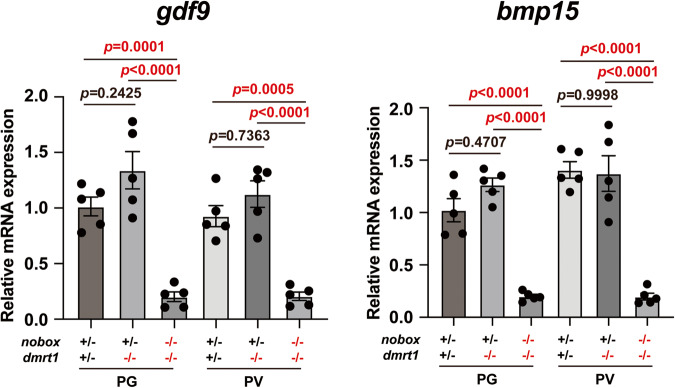
Expression of *gdf9* and *bmp15* genes in PG and PV follicles of *nobox*−/−*;dmrt1*−/− double mutant at 100 dpf. Total RNA was extracted from the isolated PG and PV follicles and reverse transcribed into cDNA for real-time PCR analysis. Each data point represents PG or PV follicles isolated and pooled from two fish for each genotype, totaling 10 fish (*n* = 5 independent samples). The mRNA levels of each target gene were normalized to that of the housekeeping gene *ef1a*, and expressed as a fold change relative to the control. Data shown are mean ± SEM, *P* values revealed by one-way ANOVA and Tukey’s test.

Nobox is an oocyte-specific transcriptional factor while estrogens are produced in the surrounding follicle cells by aromatase (*cyp19a1a*)^26,33,53^. This suggests that the reduced expression of *cyp19a1a* in *nobox* mutant must be mediated by other signaling molecules from the oocyte. As growth differentiation factor 9 (GDF9) and bone morphogenetic protein 15 (BMP15/GDF9B) are the two best characterized oocyte-derived growth factors^54^ and they both could be regulated by NOBOX in mice^26,55–57^, we hypothesized that these TGF-β family members could be potential factors that mediate Nobox regulation of aromatase expression. In support of this idea were recent studies showing downregulation of *cyp19a1a* in *bmp15* null zebrafish^58^. To provide further evidence for the possible involvement of Gdf9 and Bmp15 in Nobox regulation of *cyp19a1a*, we examined the expression of *gdf9* and *bmp15* in the double mutants (*nobox*−/−*;dmrt1*−/−) (no females available in *nobox* single mutant). The results showed that both genes were almost shut off in PG and PV follicles in the absence of *nobox* (*nobox*−/−*;dmrt1*−/−) compared to the control (*nobox*+*/−;dmrt1*+*/−*) and *dmrt1* single mutant (*nobox*+*/−;dmrt1*−/−) (Fig. 7).

## Discussion

Our previous studies showed that knocking out the oocyte-specific transcription factor *figla* or *nobox* led to an all-male phenotype in zebrafish^34,35^, suggesting crucial roles for these germ cell factors in sex differentiation and gametogenesis, especially folliculogenesis. The all-male phenotype in *figla* and *nobox* mutants has prevented us from understanding their functions in folliculogenesis due to early differentiation of the juvenile ovary to males. To explore the roles of *figla* and *nobox*, especially their differential roles in folliculogenesis, we weakened the dominance of the male-promoting pathway by deleting *dmrt1* gene, which is essential for male differentiation and spermatogenesis in vertebrates^36,59^. We have successfully used this approach to study ovarian aromatase (*cyp19a1a*), leading to the discovery that *cyp19ala* and estrogens are essential for ovarian differentiation but not for early folliculogenesis from PG to PV stage^36^. Two double mutants were created in the present study (*figla*−/−*;dmrt1*−/− and *nobox*−/−*;dmrt1*−/−) for analysis, which has further explored the differential roles of the two transcription factors in controlling folliculogenesis and their action mechanisms, especially Nobox.

Zebrafish is a juvenile hermaphrodite with all individuals forming ovary-like gonads first before developing further into functional ovaries and testes^60^. In the juvenile ovary, the germ cells initiate meiosis to become oocytes at chromatin nucleolar (CN) stage (stage Ia) and in some fish perinucleolar (PN) stage (stage Ib) as well^34,61^. The CN oocytes are clustered in germ cell cysts with synchronous development in each cyst, and some oocytes may develop into PN follicles after the process of cyst breakdown or follicle assembly to become individual follicles. How these events are regulated remains largely unknown. Our recent studies demonstrated that two oocyte-specific transcription factors, Figla and Nobox, played important roles in juvenile ovary to regulate ovarian differentiation and follicle formation.

In *figla*-null zebrafish, the germ cells of CN stage remained in cystic clusters undergoing meiosis; however, these oocyte-like cells could not form individual follicles, indicating failed cyst breakdown or follicle formation. All mutant fish turned into males quickly, generating an all-male phenotype^34^. Similar to *figla* mutant, the *nobox*-null zebrafish also showed extremely underdeveloped ovaries in juveniles; however, individual follicles of PN stage could occasionally form in the mutant, in sharp contrast to *figla*-null fish. Unfortunately, further assessment of the functional importance of Nobox in regulating subsequent follicle development was not possible due to the early and quick masculinization into males, similar to the *figla* mutant^35^. To evaluate roles especially differential roles of Figla and Nobox in controlling folliculogenesis during and after follicle assembly, we attenuated the male-promoting pathway so as to allow the mutant females of *figla* and *nobox* to fully display their defects in follicle development without early conversion to males. We have used this approach to discover that aromatase (*cyp19a1a*) and therefore estrogens are not essential for the development of follicles from PG to PV stage, in contrast to the traditional views. The loss of *cyp19a1a* gene also resulted in an all-male phenotype with PN follicles occasionally formed in the juvenile ovaries^42^, similar to the *nobox* mutant^35^. Interestingly, the ovary could form normally and folliculogenesis resumed in the double mutant of *cyp19a1a* and *dmrt1* (*cyp19a1a*−/−*;dmrt1*−/−), which prevented early sex reversal from females to males^36^.

Using the same approach, we created two double mutant fish in the present study, *figla*−/−*;dmrt1*−/− and *nobox*−/−*;dmrt1*−/−, and analyzed their folliculogenesis. The results showed that *figla*−/−*;dmrt1*−/− exhibited the same phenotype as that seen in the *figla* single mutant (*figla*−/−), *i.e*., cystic CN oocytes without formation of PN follicles. In contrast, the PN follicles could form adequately in the females of *nobox*−/−*;dmrt1*−/−. Not only could these follicles develop beyond the PG stage, but they could also progress to the PV stage, marked by the formation of cortical alveoli. This indicates a successful PG-PV transition or follicle activation. Despite these observed follicle developments, the ovaries of the double mutant were significantly smaller than those of the control, containing far fewer follicles. This reduction may be partially due to a loss of oocytes, as suggested by the presence of numerous vacuoles in the mutant ovaries. These results indicate clearly that Figla and Nobox are both important for folliculogenesis, albeit at different stages of the process. Figla primarily influences cyst breakdown and follicle formation, while Nobox acts as a promoter and stabilizer for subsequent progression of follicle development, especially the transition from PV to EV stage. This agrees well with the genetic studies in the mouse model^62,63^, suggesting functional conservation of these oocyte-specific transcription factors in vertebrates. Also, the expression of *Figla* was normal in *Nobox*-null ovary in mice and medaka;^26,27^ however, no expression of *Nobox* could be detected in the *Figla*-null gonads^27,64^. There results suggest that FIGLA might be an upstream regulator of *Nobox* expression. Alternatively, FIGLA disruption might result in a complete failure of folliculogenesis, therefore preventing *Nobox* expression. More studies are needed in different models to address this issue, including zebrafish.

Interestingly, *nobox*−/−*;dmrt1*−/− displayed a similar phenotype to that of *cyp19a1a*−/−*;dmrt1*−/− in that follicles could form and develop to PV stage with normal formation of cortical alveoli. However, the PV follicles in both double mutants could not develop further into the vitellogenic growth phase due to the lack of yolk accumulation. Since vitellogenesis in non-mammalian vertebrates is estrogen-dependent, the similarity between *nobox*−/−*;dmrt1*−/− and *cyp19a1a*−/−*;dmrt1*−/− raised an interesting question about the possible involvement of estrogens in Nobox regulation of follicle development.

To address this issue, we performed a series of experiments or analyses. First, we determined *cyp19a1a* expression in the ovary and E2 level in the serum. Both decreased significantly in *nobox*−/−*;dmrt1*−/− females compared to the control fish and *dmrt1* single mutant. In agreement with this was the regression of the female secondary sex characteristics, genital papilla, in *nobox*−/−*;dmrt1*−/− females. The most direct and conclusive evidence for roles of estrogen signaling in Nobox actions was the observation that treatment with E2 could rescue the phenotypes of not only *nobox*−/−*;dmrt1*−/− but also *cyp19a1a*−/−*;dmrt1*−/−. The PV follicles in both mutants resumed vitellogenesis by accumulating yolk mass in the oocytes in response to E2 supplementation.

Interestingly, although E2 treatment could rescue vitellogenic growth in the double mutant *nobox*−/−*;dmrt1*−/−, the follicles could only develop maximally to the MV stage, in contrast to the double mutant *cyp19a1a*−/−*;dmrt1*−/−, whose follicles could develop to the final FG stage in response to E2 and undergo oocyte maturation and ovulation. The blockade of follicle development at MV stage or MV-LV transition in *nobox*−/−*;dmrt1*−/− after E2 treatment suggests that in addition to regulating *cyp19a1a*, Nobox may also control other regulatory factors, which are essential for follicle development beyond the MV stage. The identity of these factors remains unknown. Interestingly, similar follicle arrest at MV stage has also been reported in *bmpr2b* mutant as well as the double mutants *gdf9*−/−*;inha*−/− and *bmp15*−/−*;inha*−/−^52,65,66^, suggesting potential roles for BMP family members in promoting follicle growth at the MV stage. This would be an interesting issue to explore in future studies.

Since *nobox* and *cyp19a1a* are expressed in two different compartments of the follicle, namely oocyte and follicle cells respectively, it is unlikely that Nobox could directly regulate *cyp19a1a* expression. Instead, the regulation is likely mediated by factors secreted by the oocyte. Although the identity of such factors remains unknown, the potential candidates include Gdf9 and Bmp15, which are the two best characterized oocyte-specific growth factors in mammals^54,67^. To provide supportive evidence for this, we examined the expression of *gdf9* and *bmp15* in the ovary of *nobox*−/−*;dmrt1*−/−. The data showed that the expression of these two genes was almost shut off in the mutant ovary. Further studies are needed to verify their roles in mediating Nobox control of *cyp19a1a* expression.

**Fig. 8:**
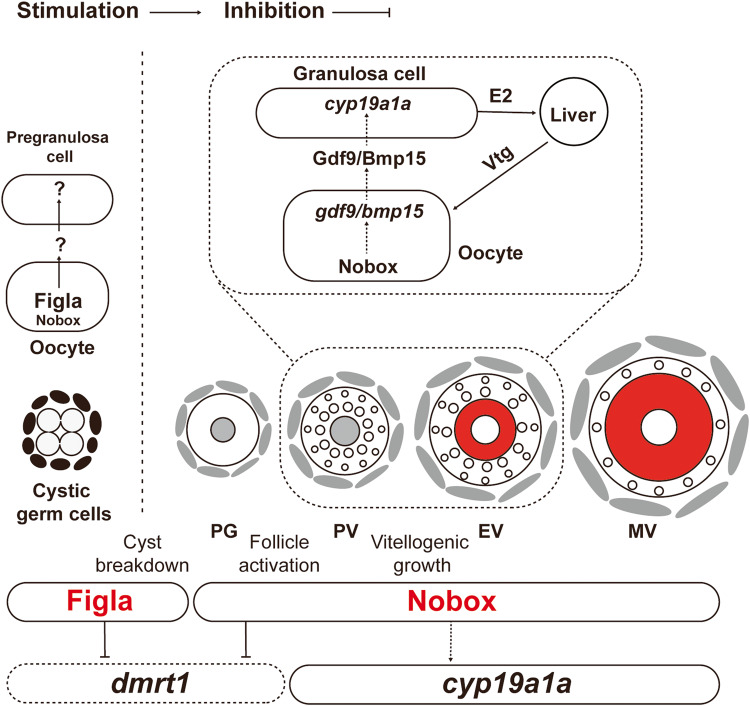
A working model on differential roles of Figla and Nobox in zebrafish folliculogenesis. Figla plays a critical role in controlling follicle formation in the event of cyst breakdown or follicle assembly, while Nobox is more involved in regulating follicle development after its formation, including such events as follicle activation (PG-PV transition) and vitellogenic growth (PV-EV transition). Nobox controls vitellogenic growth by regulating aromatase (*cyp19a1a*) expression in the follicle cells, which may be mediated by oocyte-secreted signaling molecules such as Gdf9 and Bmp15.

In mammals, Nobox has been reported to be a potential transcription factor to regulate the expression of GDF9 and BMP15. Knockout of *Nobox* gene in mice resulted in a complete loss of expression of both *Gdf9* and *Bmp15* together with other oocyte-specific genes^26^. NOBOX binding elements (NBEs) have been identified in the promoter region of mouse *Gdf9* gene and their functionality has been confirmed by luciferase reporter and ChIP assays^56^. As for BMP15, although its expression was shut off in *Nobox*-null mice^26^, direct evidence is needed to support NOBOX regulation of *Bmp15* expression at the transcription level. Our data in the present study also implicate Gdf9 and Bmp15 in mediating Nobox regulation of the follicle cells. Both genes were significantly downregulated in the double mutant ovary (*nobox*−/−*;dmrt1*−/−). Further evidence for the role of Bmp15 in relaying signals of oocyte specific Nobox to the surrounding follicle cells came from recent studies on *bmp15* in zebrafish. Knockout of zebrafish *bmp15* gene resulted in a complete arrest of follicles at PV stage with normal formation of cortical alveoli but no yolk accumulation^52,58^. Our genetic and pharmacological experiments provided clear evidence that such arrest of follicle development in *bmp15*-null zebrafish was due to reduced expression of *cyp19a1a* and therefore estrogen production^52^. However, more evidence is needed to confirm the regulation of Gdf9 and Bmp15 (or other molecules) by Nobox and their roles in mediating oocyte regulation of follicle cell functions including *cyp19a1a* expression and estrogen biosynthesis (Fig. 8).

In summary, we performed genetic analysis for roles of oocyte-specific transcription factors *figla* and *nobox* in zebrafish folliculogenesis. By attenuating the male-promoting pathway via deleting *dmrt1* gene, we were able to evaluate the functions of both Figla and Nobox in controlling folliculogenesis without being interrupted by early sex reversal to males. Our data confirmed that Figla is essential for germ cell cyst breakdown or follicle assembly whereas Nobox is important for the subsequent progression of follicle development, especially the transition from previtellogenic stage to vitellogenic growth (PV-EV transition). We provided further evidence that the Nobox-dependent vitellogenic growth was due to a deficiency in estrogen production. This study has provided strong evidence in a fish model for the important roles of oocyte in orchestrating folliculogenesis (Fig. 8).

## Methods

### Animal

The mutant zebrafish lines for *cyp19a1a* (umo5), *figla* (umo14), *dmrt1* (umo15), *nobox* (umo36) were produced in our previous studies^34–36,42^. The fish were reared at 28 ± 1 °C with a photoperiod of 14-h light and 10-h dark in a flow-through aquarium system (Tecniplast, Buguggiate, Italy). The larvae were reared in nursery tanks with paramecia and artemia before transfer to the aquarium system, and the adults were fed with artemia and Otohime fish diet (Marubeni Nisshin Feed, Tokyo, Japan). Zebrafish husbandry and all experiments were conducted in full accordance with animal care and use guidelines with ethical approval by the Research Ethics Committee of the University of Macau (Approval. No. AEC-13-002).

### Genotyping

Genomic DNA from an embryo or a small piece of the caudal fin was extracted by alkaline lysis^68^. The sample was incubated in 30–50 µl NaOH (50 nmol/µl) at 95°C for 10 min to extract the genomic DNA. Then, 3–5 µl Tris-HCl (pH 8.0) was added for neutralization. The extract was used for high-resolution melting analysis (HRMA), and the melt curves were analyzed with the Precision Melt Analysis software (Bio-Rad, Hercules, CA)^68^. The primers used for genotyping are listed in Supplementary Table 1.

### Sampling and histological examination

The fish were sampled at different time points for phenotype analysis. The fish were sacrificed after anaesthetization with MS222 (Sigma, St. Louis, MO). The gross morphology of each fish was photographed with a digital camera (Canon EOS 700D). The pectoral fins and cloaca of each sampled fish were examined on the Nikon SMZ18 dissecting microscope (Nikon, Tokyo, Japan) for breeding tubercles and genital papilla and photographed with the Digit Sight DS-Fi2 camera (Nikon).

For histological analysis, the entire fish were fixed in Bouin’s fixative for at least 24 h. Dehydration and infiltration were then performed on the ASP6025S Automatic Vacuum Tissue Processor (Leica, Wetzlar, Germany). The samples were embedded with paraffin, followed by serial sectioning at 5 µm. The sections were stained with hematoxylin and eosin (H&E) and viewed on the ECLIPSE Ni-U microscope (Nikon). The photos were taken with the Digit Sight DS-Fi2 camera (Nikon). Sibling wild type (+/+) and/or heterozygous (+/−) fish were used as controls for phenotype analysis.

### Follicle staging

Follicles were staged according to size and morphological features such as cortical alveoli and yolk granules as reported^69–71,72^. We divide follicles into six stages: primary growth (PG, <150 µm), previtellogenic (PV, ~250 µm), early vitellogenic (EV, ~350 µm), mid-vitellogenic (MV, ~450 µm), late vitellogenic (LV, ~550 µm) and full-grown (FG, >650 µm).

### Fertility assessment

Female fertility was assessed by the number of eggs spawned in natural breeding with males. The female fish from different groups were paired with wild-type (+/+) males individually for natural spawning. After spawning in the morning, the number of eggs released by each female was counted.

### Sex identification

In general, the sex was identified according to dimorphic morphological features, such as body shape, fin color, and genital papilla^73^, and, if necessary, by dissection under the Nikon SMZ18 stereomicroscope (Nikon). The sex identity was confirmed by histology at the end of sampling.

### RNA extraction and quantitative real-time PCR

Total RNA was extracted from isolated PG and PV follicles^74^ using TRIzol (Invitrogen, Waltham, MA) according to the manufacturer’s protocol. Reverse transcription was performed using M-MLV reverse transcriptase (Invitrogen). Real-time PCR was performed on the CFX384 Real-Time System (Bio-Rad) using primers listed in Supplementary Table 1. Melting curve analysis was performed to demonstrate primer specificity. A standard curve was included in each PCR assay for quantification. The relative gene expression level was normalized to the level of *ef1a* in each sample and expressed as fold change compared to the control group.

### Measurement of serum E2

After anaesthetization with MS222, the blood was gently collected from the heart of each fish using a 10-μL tip and transferred into a 1.5-mL tube. The samples were left at room temperature for 1 h to separate the serum. The supernatants were collected after centrifugation (3000 rpm, 30 min, 4 °C). The levels of E2 in the serum were measured using an ELISA kit (Neogen Corporation, Lansing, MI; RRID:AB_2935669) according to the manufacturer’s instructions.

### Oral administration of E2

Different groups of females were treated with E2 for 15 days from 70 to 85 dpf. In brief, the fish were fed twice a day with the E2-containing Otohime fish diet (0 or 2 μg/g), each at 5% of the total body weight in the tank (10% per day in total). During the treatment period, the water was renewed daily to maintain good water quality.

### Statistics and Reproducibility

All values are presented as the mean ± sem. The data were statistically analyzed by Student’s t-test or one-way ANOVA using Prism 9 (GraphPad Prism, San Diego, CA). The significance is shown by the *P* value in the figures (*P* > 0.05 or ns, not significant). The sample sizes represent independent biological replicates. All experiments were performed at least twice.

### Reporting summary

Further information on research design is available in the Nature Portfolio Reporting Summary linked to this article.

### Supplementary information

Supplementary information

Supplementary Data 1

Reporting Summary

## Data Availability

Original data generated or analyzed during this study are included in this published article or in the data repositories listed in References. Supplementary Data 1 contains all individual data values plotted in the figures, whereas Supplementary Table 1 contains all primers used in this study.
