# Publisher Correction: Genetic evidence for differential functions of *figla* and *nobox* in zebrafish ovarian differentiation and folliculogenesis

**DOI:** 10.1038/s42003-023-05651-y

**Published:** 2023-12-13

**Authors:** Kun Wu, Yue Zhai, Mingming Qin, Cheng Zhao, Nana Ai, Jianguo He, Wei Ge

**Affiliations:** 1 Department of Biomedical Sciences and Centre of Reproduction, Development and Aging (CRDA), Faculty of Health Sciences, University of Macau, 999078 Taipa, Macau China; 2 School of Marine Sciences, Sun Yat-sen University, 519082 Zhuhai, China; 3 Southern Marine Sciences and Engineering Guangdong Laboratory (Zhuhai), 519082 Zhuhai, China

**Keywords:** Oogenesis, Development

Correction to: *Communications Biology* 10.1038/s42003-023-05551-1, published online 21 November 2023.

The original version of the Article contained errors in the order of the references numbered 72, 73 and 74. The in-text citations were correctly ordered. The reference list should read:

72. Li, C. W. & Ge, W. Spatiotemporal expression of bone morphogenetic protein family ligands and receptors in the zebrafish ovary: a potential paracrine signaling mechanism for oocyte-follicle cell communication. *Biol. Reprod*. **85**, 977–986 (2011).

73. Brion, F. et al. Impacts of 17 beta-estradiol, including environmentally relevant concentrations, on reproduction after exposure during embryolarval-, juvenile- and adult-life stages in zebrafish (Danio rerio). *Aquat. Toxicol*. **68**, 193–217 (2004).

74. Zhu, B., Pardeshi, L., Chen, Y. & Ge, W. Transcriptomic analysis for differentially expressed genes in ovarian follicle activation in the zebrafish. *Front. Endocrinol. (Lausanne)* **9**, 593 (2018).

This has now been corrected in the PDF and HTML versions of the Article.

