# Supplementary material for: Genetic evidence for differential functions of figla and nobox in zebrafish ovarian differentiation and folliculogenesis
Source: Commun Biol. 2023 Nov 21;6:1185. doi: 10.1038/s42003-023-05551-1 (PMC10663522; doi:10.1038/s42003-023-05551-1)
Supplement: Supplementary file 1 — Supplementary information [file 42003_2023_5551_MOESM1_ESM.pdf]

Supplementary information

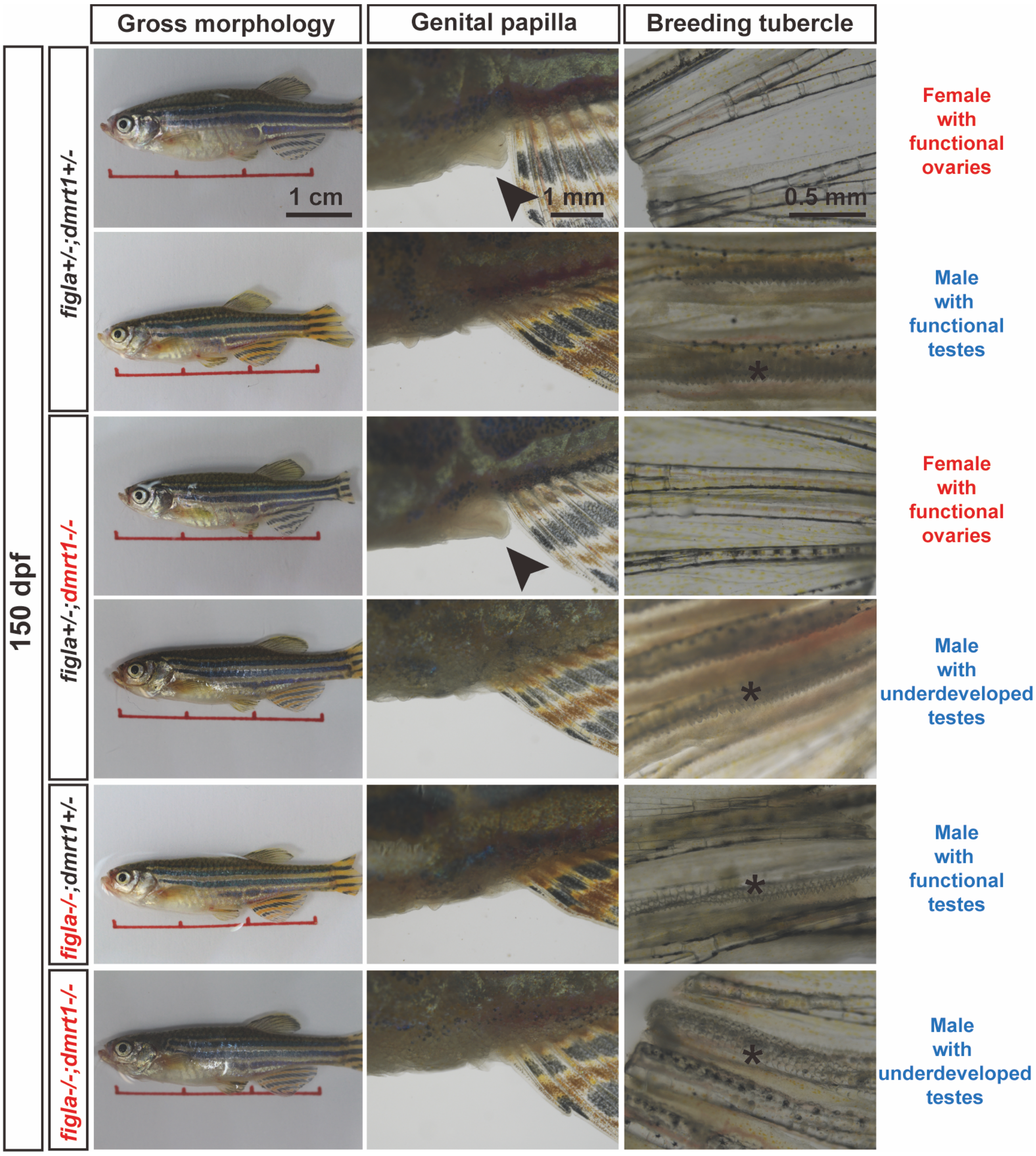

Supplementary Figure 1: Phenotype analysis of different genotypes of *figla* and *dmrt1* mutations (n=3 per fish) at 150 dpf. Gross morphology and secondary sex characteristics of four different genotypes. Asterisk, breeding tubercles; arrowhead, genital papilla.

Table 1. Primers Sequence

Gene	Primer	Sequence (5' to 3')	application
dmrt1	*dmrt1-F*	CCGGTTCAGACACTCTGGTG	HRMA
dmrt1	*dmrt1-R*	CCTCACCTAGGTTGGTTGGAG	HRMA
cyp19a1a	*cyp19a1a-F*	TGGGTCCTGTTGTCTCCTAC	HRMA
cyp19a1a	*cyp19a1a-R*	TGATCCAGACACGCACAATG	HRMA
nobox	*nobox-F*	CCTCCTCTAGAAGACGACTGT	HRMA
nobox	*nobox-R*	GGGATGGCATAACAAAAGGTCG	HRMA
figla	*figla-F*	CCGGCAAAAAGTTCAGAGCC	HRMA
figla	*figla-R*	GGTTCAGCACTTTCACGCAG	HRMA
gdf9	*gdf9-F*	GAGTCTGTTGAACCCGACG	qPCR
gdf9	*gdf9-R*	GCAGGTGGATGTCCTTCTTA	qPCR
bmp15	*bmp15-F*	TGGGTCCAACACCATAAGACTG	qPCR
bmp15	*bmp15-R*	GACGCCTTCACCAGTTTGTC	qPCR
ef1a	*ef1a-F*	GGCTGACTGTGCTGTGCTGATTG	qPCR
ef1a	*ef1a-R*	CTTGTCGGTGGGACGGCTAGG	qPCR
cyp19a1a	*cyp19a1a-1*	TGTGCGTGTCTGGATCAATGG	qPCR
cyp19a1a	*cyp19a1a-2*	AAGCCCTGGACCTGTGAGAG	qPCR

Forward (F) and Reverse (R) primers used to amplify the genomic regions surrounding targeted sequences of the *dmrt1*, *nobox*, *figla*, *cyp19a1a*, *gdf9*, *bmp15* and *ef1a* loci.
